# Face tuning in female and male individuals with depression

**DOI:** 10.1192/j.eurpsy.2021.1830

**Published:** 2021-08-13

**Authors:** J. Kubon, A. Sokolov, R. Popp, A. Fallgatter, M. Pavlova

**Affiliations:** Psychiatry And Psychotherapy, Eberhard Karls Universitaet Tuebingen, Tuebingen, Germany

**Keywords:** face pareidolia, social cognition, Depression, gender impact

## Abstract

**Introduction:**

The current COVID-19 pandemic brings social isolation to our daily lives that may elevate depression. The impact of major depressive disorder (MDD) on social cognitive functioning is far from understood, but essential for prevention and treatment of this neuropsychiatric condition.

**Objectives:**

Our aim was to examine (i) whether face tuning is lower in depression; and (ii) how it is related to other cognitive abilities (such as perceptional organization). Furthermore, we intended to clarify gender impact on face tuning in MDD, as twice more females are affected.

**Methods:**

Using a recently developed paradigm, the Face-n-Food task, we examined face tuning in 26 patients with MDD and 26 person-by-person matched controls. The advantage of non-face images is that its single elements do not promote face processing.

**Results:**

Strikingly, MDD individuals showed intact face tuning. As sex ratio in our patient sample was about 2:1 (as in MDD population in general), we recruited additional male patients and found that MDD male patients were as good as female patients. Yet, while face tuning in MDD patients showed a significant correlation with perceptual organization abilities, in controls, it was linked with social cognition.

**Conclusions:**

The outcome suggests that the origins of aberrant social functioning in MDD lie in maladaptive cognitive schemas rather than in a lack of sensitivity towards social signals per se. To elucidate neural circuits involved in face tuning in MDD, a magnetoencephalography (MEG) study with the Face-n-Food images is currently under progress.

**Disclosure:**

No significant relationships.

